# Prediction of acute kidney injury after noncardiac surgery in intensive care unit by machine learning

**DOI:** 10.1097/MS9.0000000000003699

**Published:** 2025-08-12

**Authors:** Jianxiao Chen, Tianyi Ai, Zishu Song, Weijun Zhang, Ting Yang, An Shi, Jiajia Tang, Peng Shao, Weiyan Zheng, Zhiqiang Shi, Mingli Zhu

**Affiliations:** Department of Critical Care Medicine, Renji Hospital, School of Medicine, Shanghai Jiao Tong University, Shanghai, China

**Keywords:** acute kidney injury, intensive care unit, machine learning, noncardiac surgery, prediction model

## Abstract

**Background:**

The study aimed to develop multiple machine learning models to predict acute kidney injury after noncardiac surgery (NCS-AKI) and screen for the best predictive model.

**Method:**

A total of 778 patients admitted to the surgical intensive care unit (SICU) were screened from July 2022 to December 2022. Six machine algorithms were used to predict early postoperative AKI.

**Results:**

A total of 704 patients were enrolled, 191 (27.1%) presented with early AKI. The survival rate of the AKI patients within 28 days was lower than that of the non-AKI (87.9% vs. 96.3%, log-rank test: *P* < 0.001). Among the six prediction models, the support vector machine (SVM) model had the best prediction performance, with an AUC value of 0.81 for the test set and the best net benefit compared with the other tools. The calibration curve of the model had a *P*-value of 0.68, indicating good fitness. The decision curve of the model indicates that over 80% of the model’s predictions resulted in benefits.

**Conclusion:**

NCS-AKI had a high incidence in the SICU, and AKI patients had a lower 28-day survival rate than non-AKI patients do. The SVM had a better ability to predict early postoperative AKI.

## Introduction

Acute kidney injury (AKI) is a common complication of patients undergoing major surgery and is associated with both short-term morbidity and mortality and longer-term adverse outcomes^[1–3]^, including a longer hospital length of stay, intensive care unit (ICU) admission, the need for prolonged mechanical ventilation, tracheostomy, discharge to a nursing facility, and higher 30-day readmission rates^[4,5]^. Recent reviews of the literature have shown AKI to be a significant risk factor for chronic kidney disease^[6,7]^. AKI is also known to induce damage to distant organs, which in turn contributes to morbidity and mortality^[8]^. The incidence of AKI in surgical ICU (SICU) patients ranges from 11.8% to 30%^[4,9]^. According to data from patients admitted to the ICU following major noncardiac surgery in a cohort study, 13% of patients already had evidence of AKI at admission and 41.7% developed AKI within 48 h or less postoperatively^[10]^.

**HIGHLIGHTS**

- Cross-sectional study to predict acute kidney injury (AKI) after noncardiac surgery in intensive care unit by machine learning in single center.
- Among all 704 patients, 191 (27.1%) presented with early AKI.
- The survival rate of the AKI within 28 days was lower than that of the non-AKI (87.9% vs. 96.3%).
- The support vector machine model had a better ability to predict early postoperative AKI in the study.

AKI can be identified by the evaluation of serum creatinine (sCr) levels or urine output using the Kidney Disease Improving Global Outcomes (KDIGO) clinical practice guidelines^[11]^. However, since renal impairment typically precedes increases in creatinine, staging guidelines are only able to detect AKI after renal injury or impairment has occurred. Recently, machine learning (ML) has attracted the attention of and gained recognition from nephrologists and critical specialists because of the evolution of statistical theory and computer technology. Early prediction of AKI is important for identifying patients at risk of developing AKI and intervening early to improve outcomes. The Acute Disease Quality Initiative (ADQI) group recommended developing ML models for the early prediction of moderate to severe AKI (stage 2/3) between 48 and 72 h before diagnosis. Additionally, ADQI suggested that the prediction model should present information about patient measurements contributing to these risks and provide feedback to practitioners regarding potentially actionable items^[12]^. Many studies have approached early prediction of AKI using ML algorithms, including automatic continuous random forest algorithms^[13]^, convolutional neural networks (CNNs)^[14]^, random forests (RF), support vector machines (SVMs), and artificial neural networks (ANNs)^[15]^. The use of these models would allow early interventions for those at high risk of acute kidney injury in different populations (such as general pediatric patients in critical care, patients with sepsis and adult inpatients), but models for predicting postoperative AKI are lacking, especially for AKI after noncardiac surgery (NCS)^[16–19]^.

Therefore, novel ML techniques have been used in predictive models of AKI, showing different performance than traditional logistic regression (LR) or Cox regression analyses^[20]^. It is unknown which one or more ML algorithms are superior to others for the prediction of AKI after surgery. The present study aimed to develop multiple ML models to predict postoperative AKI earlier when patients were admitted to the SICU 48 h or less and to evaluate the predictive performance of these models.

## Materials and methods

### Patients

The study population included all adult surgical patients admitted to the SICU between 1 July 2022 and 31 December 2022. The inclusion criterion was adult patients over 18 years of age who underwent noncardiac surgery and were admitted to the SICU. The exclusion criteria were a length of stay in the ICU of less than 24 h, nonsurgical reasons for ICU admission, and end-stage renal disease (ESRD) requiring dialysis. The cross-sectional study has been reported in line with the STROCSS criteria^[21]^.

### Data collection

The collected data were divided into preoperative, intraoperative, and postoperative variables. Preoperative variables included demographic information, comorbid conditions, laboratory measurements, mean arterial pressure (MAP), Glasgow Coma Scale (GCS) score, and Acute Physiology and Chronic Health Evaluation II (APACHE II) score. Intraoperative parameters included surgical duration, fluid management, and urine output. The postoperative variable was the level of lactate. Other major variables included perioperative medications (such as vasoactive agents, mannitol, and contrast agents), the surgical department, the operating room (general room and digital subtraction angiography [DSA]), and the timing of the operation (emergency or elective operation).

### AKI definitions

The definitions for AKI were as follows and according to the KDIGO creatinine-based criteria from the date of surgery^[11]^. Stage 1 was defined as: a rise in sCr by at least 26.4 μmol/l (0.3 mg/dl) within 48 h or less, an increase to 1.5–1.9 times the baseline level; or urine output of less than 0.5 ml/kg/h for more than 6 h. Stage 2 was defined as: a rise in sCr by 2.0–2.9 times the baseline level, or urine output of less than 0.5 ml/kg/h for more than 12 hours. Stage 3 was defined as: a rise in sCr three times the baseline level, an increase to at least 354 μmol/l (4 mg/dl) with an acute increase of at least 44 μmol/l (0.5 mg/dl), treatment with renal replacement therapy (RRT) regardless of the stage at the time of RRT, urine output of less than 0.3 ml/kg/h for 24 h or more, or anuria for 12 h.

### Statistical analysis

#### Data analysis

The data are presented as percentages, means ± standard deviations (SDs), or medians with interquartile ranges (IQRs) of 25% and 75%, as appropriate. Chi-squared analysis was used to compare categorical data, whereas Student’s *t*-test was used to compare continuous data. In cases where the data did not follow a normal distribution, the Mann–Whitney U-test was used. Stepwise logistic regression analysis was conducted to identify risk factors. Kaplan-Meier survival analysis was performed for the 28-day survival rate, and the log-rank test was used to compare the AKI and non-AKI groups. A significance level of *P* < 0.05 was considered statistically significant. All analyses were performed using the “R” language (version 4.1.0).

#### Predictive model and data split

After screening the feature variables using univariate regression analysis, the dataset was randomly divided into a training set (80%) and a test set (20%). Six machine learning algorithms, including Gaussian NB (Gnb), extreme gradient boosting (XGBoost), LR, RF, decision tree (DT), and SVM, were used in the training set for predicting NCS-AKI^[22]^. The machine learning model with the best predictive performance was selected to complete the external validation of the test set. The SHapley Additive exPlanations (SHAP) algorithm was used to interpret the model value. The performance of each model was compared by using the area under the receiver operating characteristic curve (AUROC), and the model with the highest area under the curve (AUC) was selected as the optimal model for each algorithm.

## Results

### The proportion of early postoperative AKI

**Figure 1.**
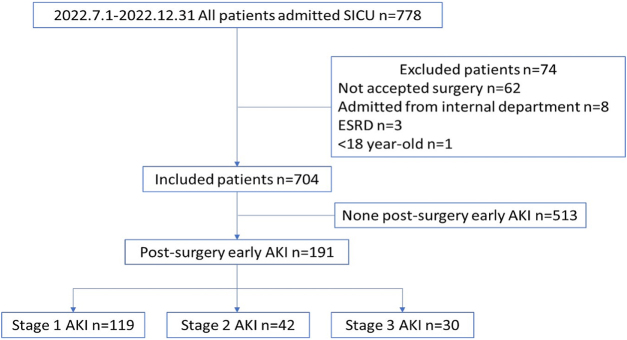
Flowchart of post-operative AKI in SICU.

A total of 778 patients who were admitted to the SICU were screened between 1 July 2022 and 31 December 2022. Among these, 74 patients were excluded from the analysis for various reasons. The exclusion criteria included 70 patients who did not undergo surgery, three patients with ESRD, and one patient who was younger than 18 years. The remaining 704 patients were included in the analysis. Among them, 191 patients (23%) developed postoperative AKI. Stage 1 AKI was observed in 119 patients (60%), stage 2 AKI in 42 patients (24%), and stage 3 AKI in 30 patients (16%). Additionally, two patients required RRT (Fig. 1).

All subjects were divided into three groups according to the preoperative sCr values. Among them, 19 cases were sCr ≥132.6 µmol/L, five cases were in stage 3 AKI, seven cases were in stage 2 AKI, six cases were in stage 1 AKI, and one case was non-AKI; Among the 65 cases with 88.4 ≤ sCr<132.6 µmol/L, six cases were in stage 3 AKI, four cases were in stage 2 AKI, 36 cases were in stage 1 AKI, 19 cases were non-AKI; There were 615 cases in sCr <88.4 µmol/L group, including 19 cases of stage 3 AKI, 31 cases of stage 2 AKI, 77 cases of stage 3 AKI, and 488 cases of non-AKI.

### Surgical department distribution of patients

In terms of surgical type, there were 247 (35.1%) cases in general surgery, 329 (46.7%) in neurosurgery, 39 (5.5%) in thoracic surgery, 39 (5.5%) in orthopedics, the rest of them in other department including urology, obstetrics, and gynecology, etc. Furthermore, 404 patients (57.2%) underwent elective surgery, whereas 300 patients (42.3%) required emergency operations. They were performed in two different operating rooms, with 550 cases (78.1%) conducted in the general operating room and 154 cases (21.9%) performed in the DSA room (Table 1).

**Table 1:** Comparison of variables between the AKI group and the non-AKI group

	All patients, *n* = 704	AKI, *n* = 191	Non-AKI, *n* = 513	*P*
Age (years)	63.5 ± 14.3	66.2 ± 14.6	62.4 ± 14.1	0.002*
Gender (male, *n*, %)	451, 64.1%	140, 73.3%	311, 60.6%	0.002*
BMI (kg/m^2^)	23.6 ± 4	23.4 ± 4.1	23.7 ± 4	0.429
Comorbidity				
Hypertension (*n*, %)	241, 34.2%	77, 40.3%	164, 32%	0.038*
Diabetes mellitus (*n*, %)	95, 13.5%	35, 18.3%	60, 11.7%	0.022*
Chronic kidney disease (*n*, %)	54, 7.7%	48, 25.1%	6, 1.2%	<0.001*
Coronary heart disease (*n*, %)	42, 6%	19, 9.9%	23, 4.5%	0.006*
History of stroke (*n*, %)	40, 5.7%	10, 5.2%	30, 5.8%	0.755
Causes of surgery				
Intracranial vascular disease (*n*, %)	250, 35.5%	71, 37.2%	179, 34.9%	0.574
Gastrointestinal tumors (*n*, %)	123, 17.5%	29, 15.2%	94, 18.3%	0.329
TBI (*n*, %)	70, 9.9%	20, 10.5%	50, 9.7%	0.775
Hepatic/gallbladder/pancreatic carcinoma (*n*, %)	63, 8.9%	18, 9.4%	45, 8.8%	0.788
Esophagus cancer	25, 3.6%	6, 3.1%	19, 3.7%	0.72
Other (*n*, %)	173, 24.6%	47, 24.6%	126, 24.6%	0.94
Relevant surgery parameters				
Department				0.508
Neurosurgery (*n*, %)	329, 46.7%	91, 47.6%	238, 46.4%	
General surgery (*n*, %)	247, 35.1%	65, 34%	182, 35.5%	
Thoracic surgery (*n*, %)	39, 5.5%	8, 4.2%	31, 6%	
Orthopedics (*n*, %)	39, 5.5%	9, 4.7%	30, 5.8%	
Others (*n*, %)	50, 7.1%	18, 9.4%	32, 6.2%	
Timing of operation				0.004*
Emergency operation (*n*, %)	300, 42.6%	98, 51.3%	202, 39.4%	
Elective operation (*n*, %)	404, 57.4%	93, 48.7%	311, 60.6%	
Operation room				0.715
General operating room (*n*, %)	550, 78.1%	151, 79.1%	399, 77.8%	
DSA room (*n*, %)	154, 21.9%	40, 20.9%	114, 22.2%	
Intra-operation variables				
Operative time (min)	174 [119.5, 259.5]	197 [124.8, 265]	165 [116.5, 255]	0.007*
Crystal infusion (L)	1.1 [0.7, 1.7]	1.3 [1, 1.8]	1.1 [0.6, 1.6]	0.002*
Colloid infusion (L)	0.5 [0.3, 0.8]	0.5 [0.5, 0.8]	0.5 [0.3, 0.8]	<0.001*
After admitted ICU variables				
APACHE II	12.6 ± 6.4	14.3 ± 6.7	12 ± 6.1	<0.001*
MAP (mmHg)	92.7 ± 13.5	90.4 ± 13.6	93.6 ± 13.4	0.004*
GCS	12.9 ± 3.6	11.7 ± 4.3	13.1 ± 3.1	<0.001*
Laboratory variables				
WBC count (×10^9^/L)	10.1 ± 4.5	10.5 ± 5	9.9 ± 4.2	0.148
Hemoglobin (g/dl)	11.8 ± 2.3	11.2 ± 2.5	11.9 ± 2.2	0.001*
Platelet (×10^9^/L)	192 ± 84.6	183.1 ± 81.3	195.3 ± 85.6	0.088
CRP (mg/L)	4.5 [1.1, 16.2]	8.3 [2.7, 36.3]	3.14 [0.9, 12.3]	<0.001*
PCT (ng/ml)	0.06 [0.03, 0.15]	0.09 [0.05, 0.6]	0.05 [0.03, 0.1]	<0.001*
D-dimer (µg/ml)	1 [0.4, 2.1]	1.24 [0.7, 2.7]	0.81 [0.3, 1.9]	<0.001*
Albumin (g/L)	35.6 ± 6.5	33.4 ± 6.6	36.4 ± 6.3	<0.001*
Total bilirubin (mmol/L)	15 [10.9, 20.4]	15 [11, 21]	14.8 [10.9, 20]	0.349
sCr (µmol/L)	62 [52, 74]	71 [56, 96]	60 [51, 69.8]	<0.001*
BUN (mmol/L)	5.7 ± 2.2	6.9 ± 2.5	5.3 ± 1.9	<0.001*
Lactate (mmol/L)	2.1 [1.5, 3]	2.4 [1.8, 3.4]	1.9 [1.4, 2.8]	<0.001*
Medicine used in peri-operation				
Vasoactive agent (*n*, %)	102, 14.5%	35, 18.3%	67, 13.1%	0.078
Contrast agent (*n*, %)	231, 32.8%	82, 42.9%	149, 29%	<0.001*
Mannitol (*n*, %)	181, 25.7%	59, 30.9%	122, 23.8%	0.055

AKI: Acute kidney injury; APACHE II: Acute Physiology and Chronic Health Evaluation II; BMI: Body mass index; BUN: blood urea nitrogen; CRP: C-reactive protein; DSA: digital subtraction angiography; GCS: Glasgow Coma Scale; MAP: mean arterial pressure; PCT: procalcitonin; sCr: serum creatinine; TBI: traumatic brain injury.

^*^ *P* < 0.05

### Patient demographic data and baseline clinical characteristics

Among the patients in this study, the average age was 63.5 ± 14.3 years. The median BMI was 23.6 ± 4 kg/m^2^. There was a total of 451 male patients, accounting for 64% of the study population. In terms of comorbidities, 34.2% of the patients had hypertension, 13.5% had diabetes, 7.7% had chronic kidney disease (CKD), 6% had coronary heart disease, and 40 patients (5.7%) had prior stroke (Table 1).

### Survival

**Figure 2.**
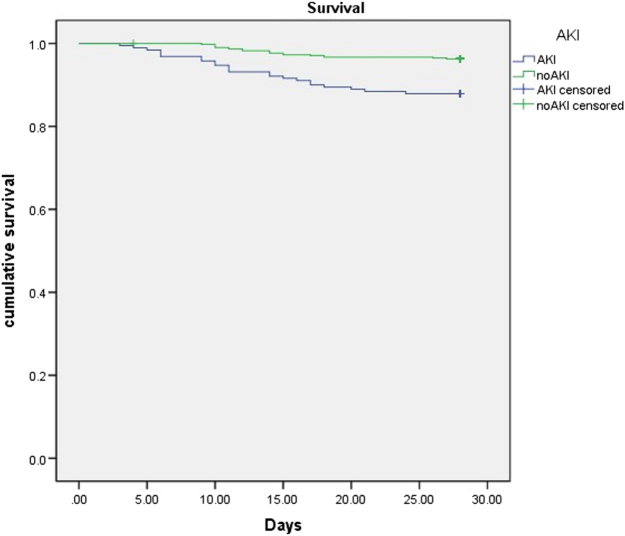
Kaplan–Meier survival curves for patients between AKI and non-AKI in SICU undergoing noncardiac surgery.

In the AKI group, 18 patients died within a span of 28 days, whereas 22 patients died in the non-AKI group. The survival rate of the AKI group within 28 days was lower than that of the non-AKI group (87.9% vs. 96.3%, log-rank test: *P* < 0.001) (Fig. 2).

### Comparison of variables between the AKI group and the non-AKI group

#### Demographics and severity of disease

Compared with non-AKI group, the AKI group had more male (73.3% vs. 60.6%, *P* = 0.002) and older (66.2 ± 14.6 vs. 62.4 ± 14.1 years, *P* = 0.002) patients. AKI group had higher APACHE II scores (14.3 ± 6.7 vs. 12 ± 6.1 *P* < 0.001) and lower GCS (11.7 ± 4.3 vs. 13.1 ± 3.1, *P* < 0.001) than the non-AKI group did. The MAP was significantly lower in AKI group than that in non-AKI group, although the MAP value was normal both in two groups (Table 1).

#### Comorbidity and causes of surgery

Compared with the non-AKI group, the AKI group had a greater prevalence of hypertension (40.3% vs. 32%, *P* = 0.038), diabetes mellitus (18.3% vs. 11.7%, *P* = 0.022), chronic kidney disease (25.1% vs. 1.2%, *P* < 0.001), and coronary heart disease (9.9% vs. 4.5%, *P* = 0.006). There was no significant difference in causes of surgery between AKI group and non-AKI group, such as intracranial vascular disease, gastrointestinal tumors, traumatic brain injury (TBI), hepatic/gallbladder/pancreatic carcinoma, esophagus cancer, and so on (Table 1).

#### Relevant surgery parameters and intra-operation variables

Compared with the non-AKI group, the AKI group had a greater proportion of patients who underwent emergency operations (51.3% vs. 39.4%, *P* = 0.004). AKI group had longer operative time and more fluid infusion during operation than non-AKI did. There was no significant difference in surgical department such as neurosurgery, general surgery, thoracic surgery, orthopedics, and so on.

#### Peri-operation medications (Table 1)

Compared with the non-AKI group, more patients received contrast agents (42.9% vs. 29%, *P* < 0.001) in AKI group. No significant difference in vasoactive agent and mannitol was shown between two groups (Table 1).

#### Laboratory variables at baseline

AKI group had higher level of C-reactive protein (CRP), procalcitonin (PCT), D-dimer, sCr, blood urea nitrogen (BUN), and lactate than non-AKI group did. Furthermore, the concentration of albumin and hemoglobin were significantly lower in the AKI group than that in the non-AKI group (Table 1).

#### Machine learning models for predicting early postoperative AKI

**Figure 3.**
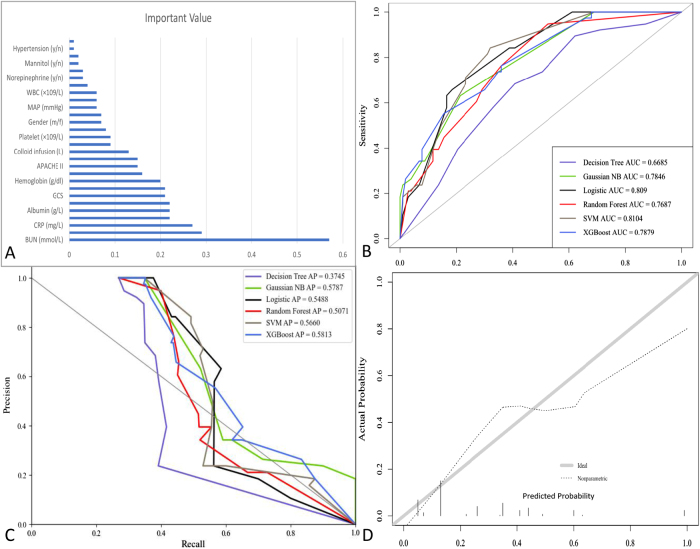
Comparison of the value of prediction among six models. (A) The result of univariate regression analysis. (B) The ROC curve of six models. (C) The precision-recall curve of six models. (D) The calibration curve of six models.

The univariate regression analysis eliminated two feature variables out of 28 before model development (Fig. 3A). Compared with the other six models, the SVM model had the best prediction performance, with an AUC value of 0.81, and the AUC value of DT, RF, Gnb, XGBoost, was 0.66, 0.76, 0.78, 0.78, 0.80, respectively. (Fig. 3B) and an ap value of 0.57 (Fig. 3C) in the test set. The calibration curve of the model demonstrated a *P*-value of 0.68, indicating good fitness (Fig. 3D).

**Figure 4.**
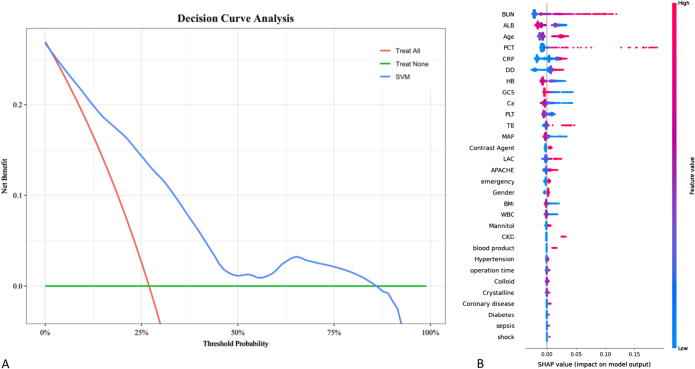
The decision curve and SHAP results of the SVM model. (A) The decision curve of the SVM model. (B) The SHAP value of each variable in SVM model. The color gradient of the point corresponded to the value of the independent variable. The importance of the independent variables is gradually lower from top to bottom. A variable value from small to large (the scatter color from blue to red) corresponded to its SHAP value from small to large, meaning that the variable had a positive relationship with AKI, the greater value, the stronger effect on AKI.

The decision curve of the model indicates that more than 80% of the model predictions result in benefits (Fig. 4A). SHAP explained the impact of different feature variables on the predicted outcome. It showed post-operative AKI was associated with higher BUN, lactate, PCT level, lower GCS, male sex, contrast agent used, larger amounts of crystalloids used intra-operatively, and previous CKD. The baseline BUN level is the most important for AKI prediction (Fig. 4B).

## Discussion

The study revealed that 27.1% of patients admitted to the SICU had postoperative AKI, and the 28-day survival rate of these AKI patients was 87.9%. Among the machine learning models for the prediction of AKI after noncardiac surgery, the SVM prediction model had the best prediction performance.

Some studies have reported varying incidence rates of AKI after surgery^[4,23–26]^. For example, Grams *et al* reported an 11.8% incidence of postoperative AKI among U.S. veterans hospitalized for surgical procedures^[4]^, whereas O’Connor *et al* reported a 13.4% incidence of AKI after major abdominal surgery^[23]^. Zarbock *et al* studied 10 568 patients from 30 countries who had undergone major surgery (postoperative ICU or high-dependency unit (HDU) admission) and reported that 1945 (18.4%) patients developed postoperative AKI (63.5% stage 1, 25.7% stage 2, 10.7% stage 3)^[24]^. Cheng *et al* reported that 5.0% of 520 707 patients in China developed postoperative AKI^[25]^. In this study, the incidence of postoperative AKI was 27.1%, which was slightly higher than that reported in the aforementioned studies and could be due to differences in surgical populations. The study data consisted mainly of ICU-admitted patients, with a greater proportion of patients who underwent abdominal surgery than the other studies had. Additionally, the number of neurosurgical patients with greater disease severity (as indicated by an average APACHE II score of approximately 12) accounted for 46.7% of the cases, potentially contributing to a higher incidence of AKI.

The present study showed the AKI group had more male (73.3%) and older patients (66.2 years old) than non-AKI group did. Several researches have reported gender and age were associated with postoperative AKI. These results showed that the risk of AKI increased by 1.1 times for each 10 years increase in age, and 1.2–-1.7 times for male patients^[4,10,24]^. Moreover, we found that the 28-day survival rate for postoperative patients with AKI was 87.9%, which was significantly greater than that for non-AKI patients and consistent with the findings of previous studies^[4,24,26]^.

ML focuses on understanding and creating learning techniques that use data to improve task performance. ML algorithms have been used in the prediction of AKI in hospitalized patients, emergency patients, and ICU patients. ML shows great promise because it integrates large amounts of data to predict AKI. To date, most prediction models have been developed for AKI following cardiac surgery^[27]^, one study has shown the models for postoperative patients which about 50% patients had accepted cardiac surgery^[28]^, but the application of prediction models for AKI after noncardiac surgery is not well known. A retrospective cohort study assessed 111,888 operations performed on adults at a single academic medical center, the model with AUROCs increased from 0.579 to 0.848 for AKI^[29]^, the cardiac surgery was accounted for 3.3%. Another retrospective single center cohort study included 50 318 surgical patients has shown the AUROCs for different models ranged between 0.797 and 0.858 for postoperative AKI, and cardiothoracic surgery accounted for 13.5%^[30]^. The present study indicates that ML is valuable for the prediction of postoperative AKI in the surgical ICU without cardiac surgery.

There is tremendous variability in research methods, which use different training variables. Extensive data, including perioperative features, are used in current prediction models^[26]^. Preoperative variables included demographic data, comorbidities, preoperative laboratory results, and operative characteristics. Intraoperative variables included colloid and crystalloid infusion, vasopressor administration, and operative time. The lactate level was used as an early postoperative variable. These data were used as different training variables, with the most commonly used variables being age, sex, diabetes status, body mass index, and surgery type^[29]^. Some studies revealed that the combination of overall perioperative variables was better for prediction than was pre- or intraoperative variables alone^[28,29,31]^.

Different mathematical models are utilized in the prediction of AKI by ML, such as LR, RF, XGBoost, DT, SVM, and NB, but the optimal model remains controversial. In the present study, the above six models were compared, and the SVM was better for the prediction of postoperative AKI than other models for this patient population. SVM is a supervised machine learning method commonly used for classification and regression problems. SVM uses a combination of several dimensions of binary classification which outperform multivariate linear regression^[32]^. Although SVM is computationally complex, it is easy to train. The advantages of SVM include excellent performance in small sample cases, good processing power for high-dimensional data, and good generalizability. Thottakkara P, *et al* have shown SVM had good performance as risk prediction model for postoperative AKI^[30]^, the same as the present study.

An important advantage of machine learning over traditional methods such as logistic regression analysis is that various machine learning algorithms do not require data to conform to statistical assumptions^[20]^. The variability of AKI machine learning predictions can be attributed in part to factors such as the specific machine learning model used, variable selection and treatment, study protocol characteristics, and the distribution of the study population.

The study has several limitations. First, this was a cross-sectional study with a relatively short time span and follow-up period; thus, the results and conclusions should be interpreted with caution. The development of prospective and controlled trials of ML for AKI prediction is needed. Second, this was a single-center study with a relatively small sample size, potentially impacting the results. Further enhancement of electronic hospital databases and increasing the number of diverse centers may improve model performance. Third, this study did not include the collection of biological markers of AKI for prediction purposes, which should be considered in future investigations.

## Conclusion

The occurrence of postoperative AKI was relatively high, and the survival rate within 28 days after surgery was lower in patients with AKI than that of non-AKI patients. The SVM had the optimal predictive performance for postoperative AKI among the six models of machine learning. In future studies, appropriate mathematical models should be selected to further develop a prediction model for AKI and provide early alert and effective prevention of postoperative AKI.

## Data Availability

All datasets generated during and/or analyzed during the current study are available upon reasonable request.

## Research registration unique identifying number (UIN)

The clinical trial has been registered at http://www.chictr.org.cn/index.aspx (ChiCTR2200055732).

## Assistance with the study

We would like to thank Tianxing Xue for his data entry.
